# Supplementary material for: Pleiotropic fitness effects of the lncRNA Uhg4 in Drosophila melanogaster
Source: BMC Genomics. 2022 Nov 30;23:781. doi: 10.1186/s12864-022-08972-0 (PMC9710044; doi:10.1186/s12864-022-08972-0)
Supplement: Supplementary file 8 — Additional file 8: Figure S1. Crossing scheme to generate Uhg4 deletion CRISPR mutants. Following injections of Cas9 and gRNA vectors into embryos of each DGRP line, resulting progeny were screened for presence of a deletion around Uhg4, and if a deletion was present, crossed to the original genetic background to generate additional flies heterozygous for the original deletion. Backcrossing siblings heterozygous for the same mutation resulted in homozygous flies that were sterile for all isolated deletion mutations. Virgin female flies heterozygous for a specific deletion were then crossed to male flies containing the CyO balancer. Resulting progeny were screened for the presence of the CyO balancer chromosome as well as the respective mutation and crossed to full siblings to establish the stock. All females were crossed as virgin flies. “Δ” refers to a specific Uhg4 deletion. [file 12864_2022_8972_MOESM8_ESM.pdf]

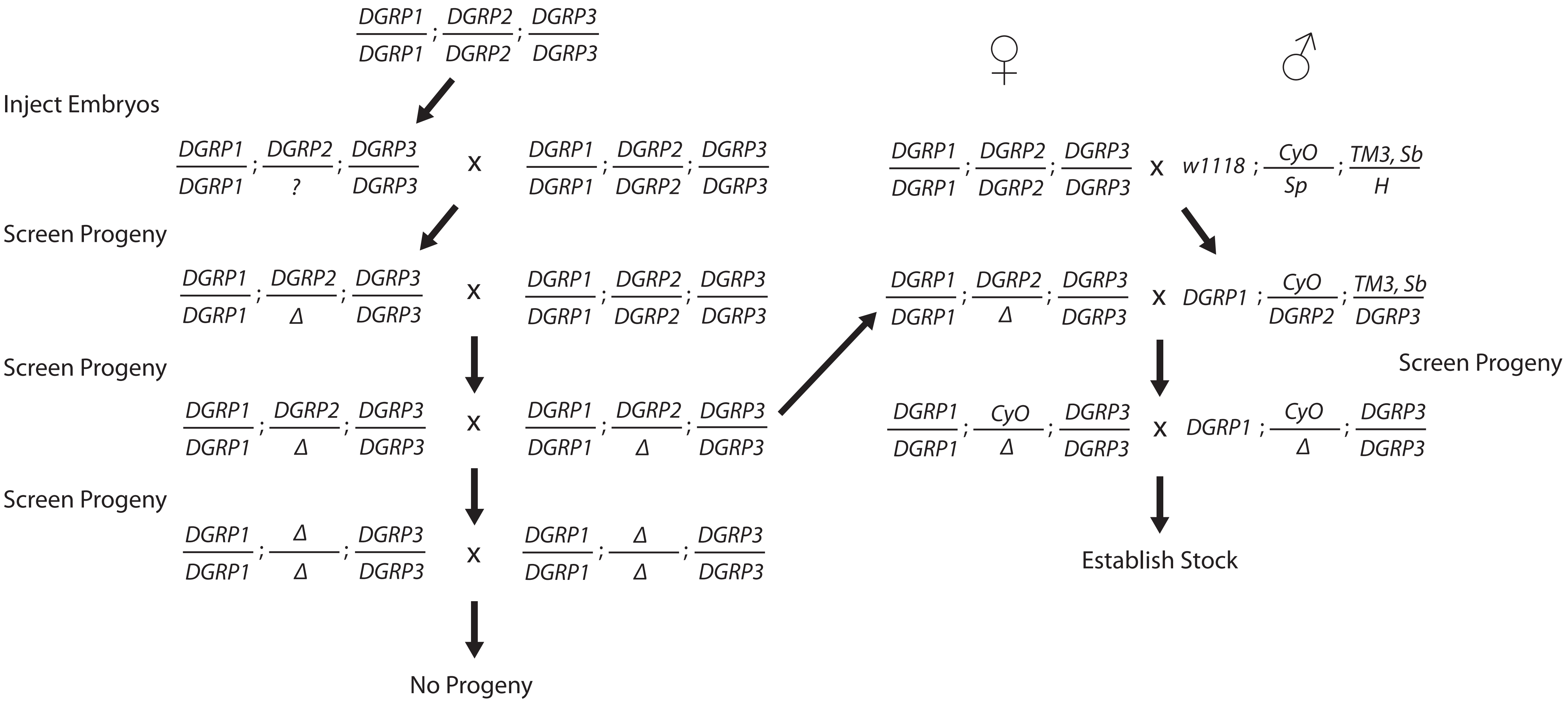

Inject Embryos
Screen Progeny
Screen Progeny
Screen Progeny
No Progeny
Screen Progeny
Establish Stock
DGRP1
DGRP2
DGRP3
w1118
CyO
Sp
TM3, Sb
H
Δ
x
