# Supplementary figures and images for: Pleiotropic fitness effects of the lncRNA Uhg4 in Drosophila melanogaster
Source: BMC Genomics. 2022 Nov 30;23:781. doi: 10.1186/s12864-022-08972-0 (PMC9710044; doi:10.1186/s12864-022-08972-0)

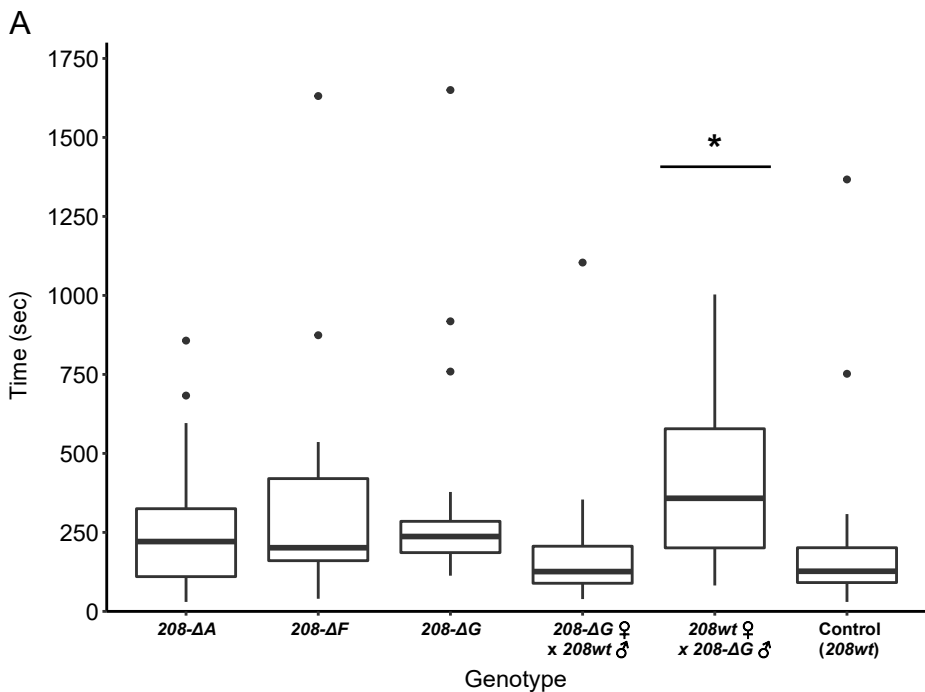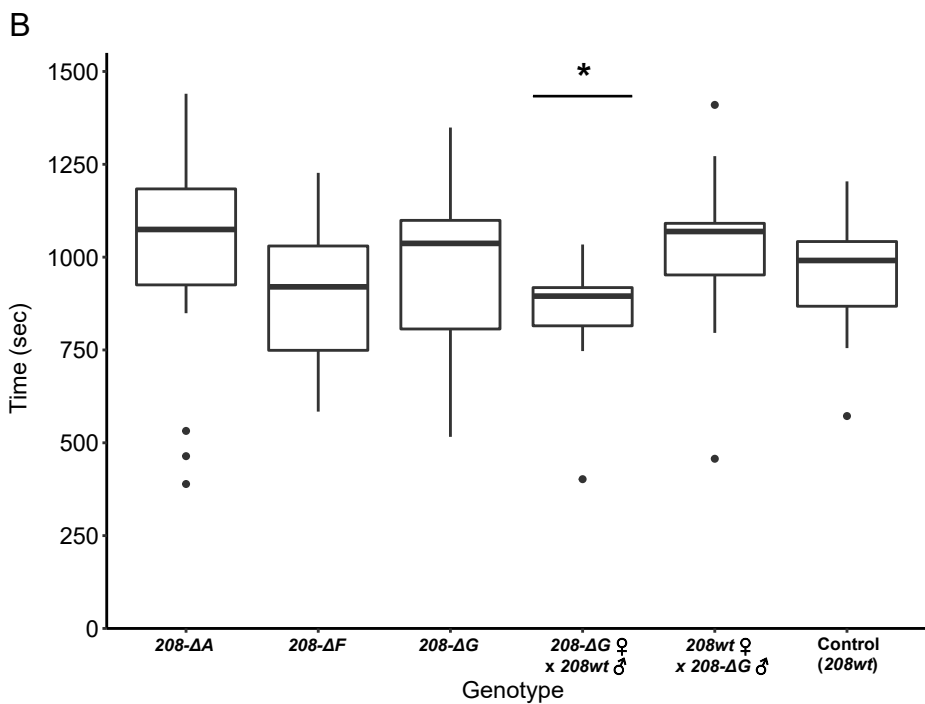

Supplement: Supplementary file 9 — Additional file 9: Figure S2. Mating phenotypes of Uhg4 deletion flies. Boxplots displaying time, in seconds, of (A) mating latency (time until mating begins) and (B) mating duration (length of copulation) for each of the Uhg4 mutant lines (208-ΔA, 208-ΔF, 208-ΔG), the control line DGRP_208 (208wt), and a representative mutant (208-ΔG) versus DGRP_208 wildtype pairings (208-ΔG females x 208wt males, 208wt females x 208-ΔG males). N = 22-24 pairings of 3-5 day old virgin flies per line. Only flies which successfully initiated or completed mating within 30 minutes were included in analysis for mating latency and mating duration, respectively. See Table S1A. * p<0.05 [file 12864_2022_8972_MOESM9_ESM.pdf]

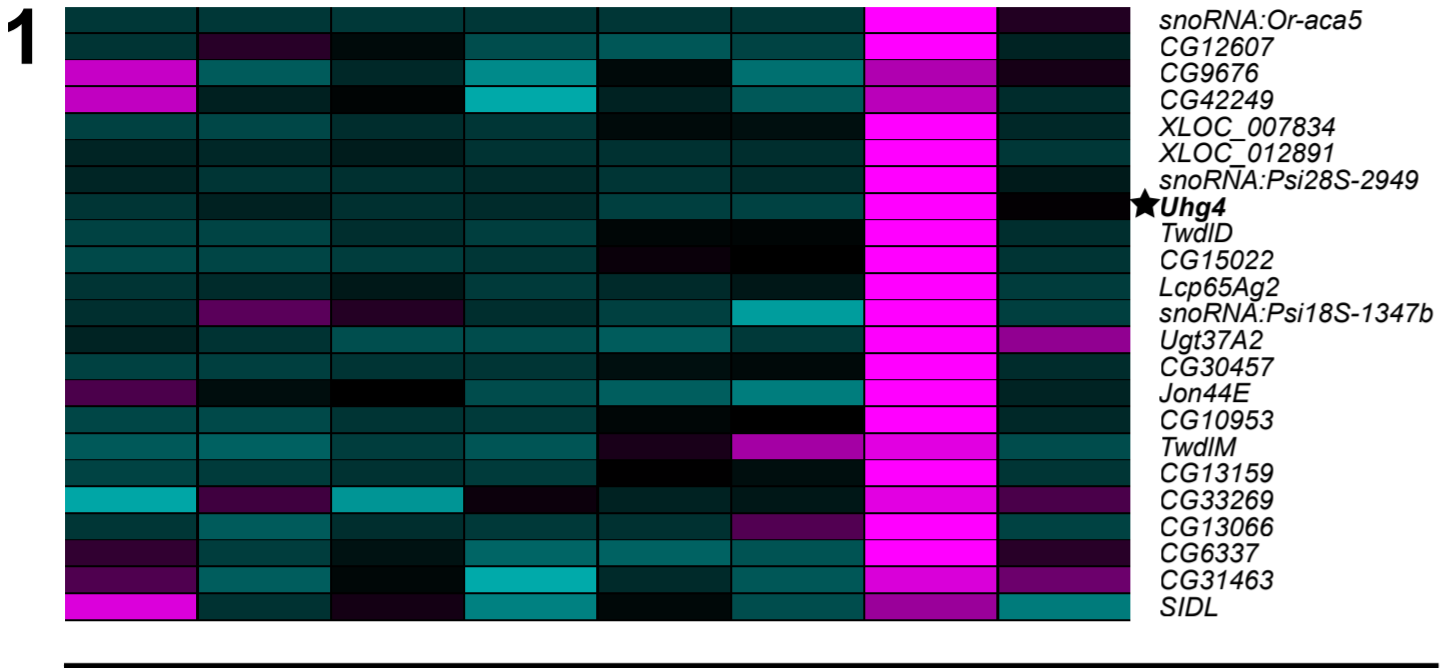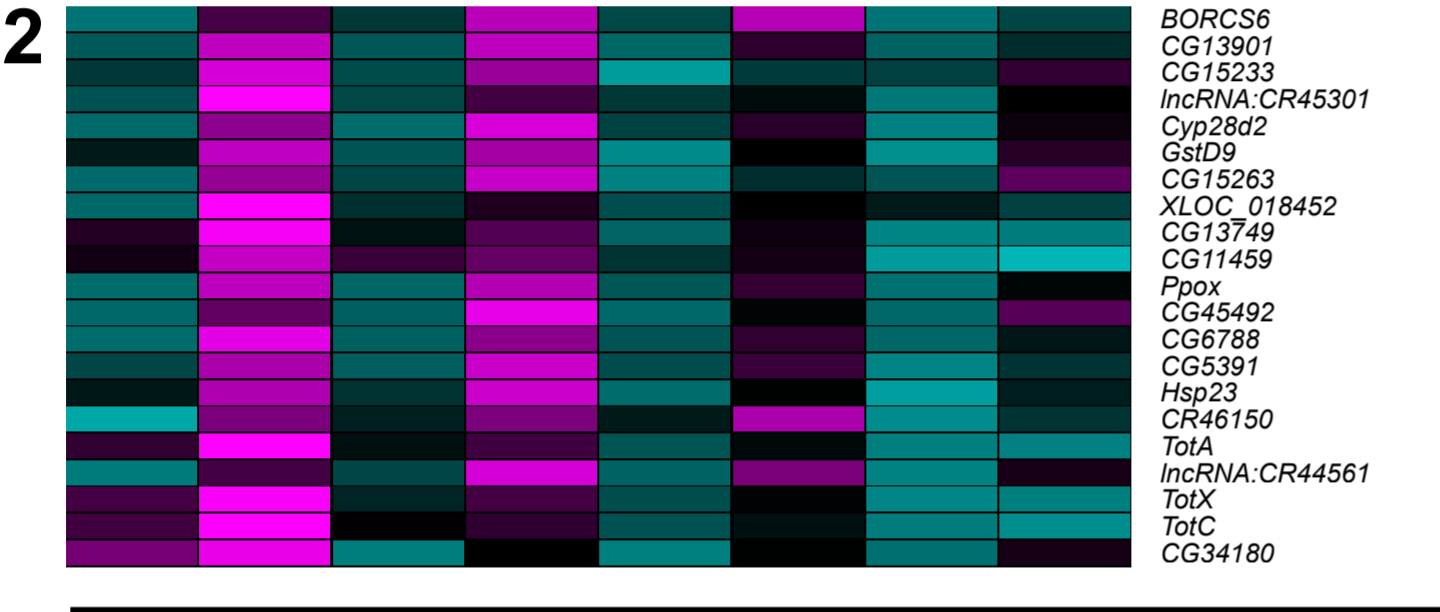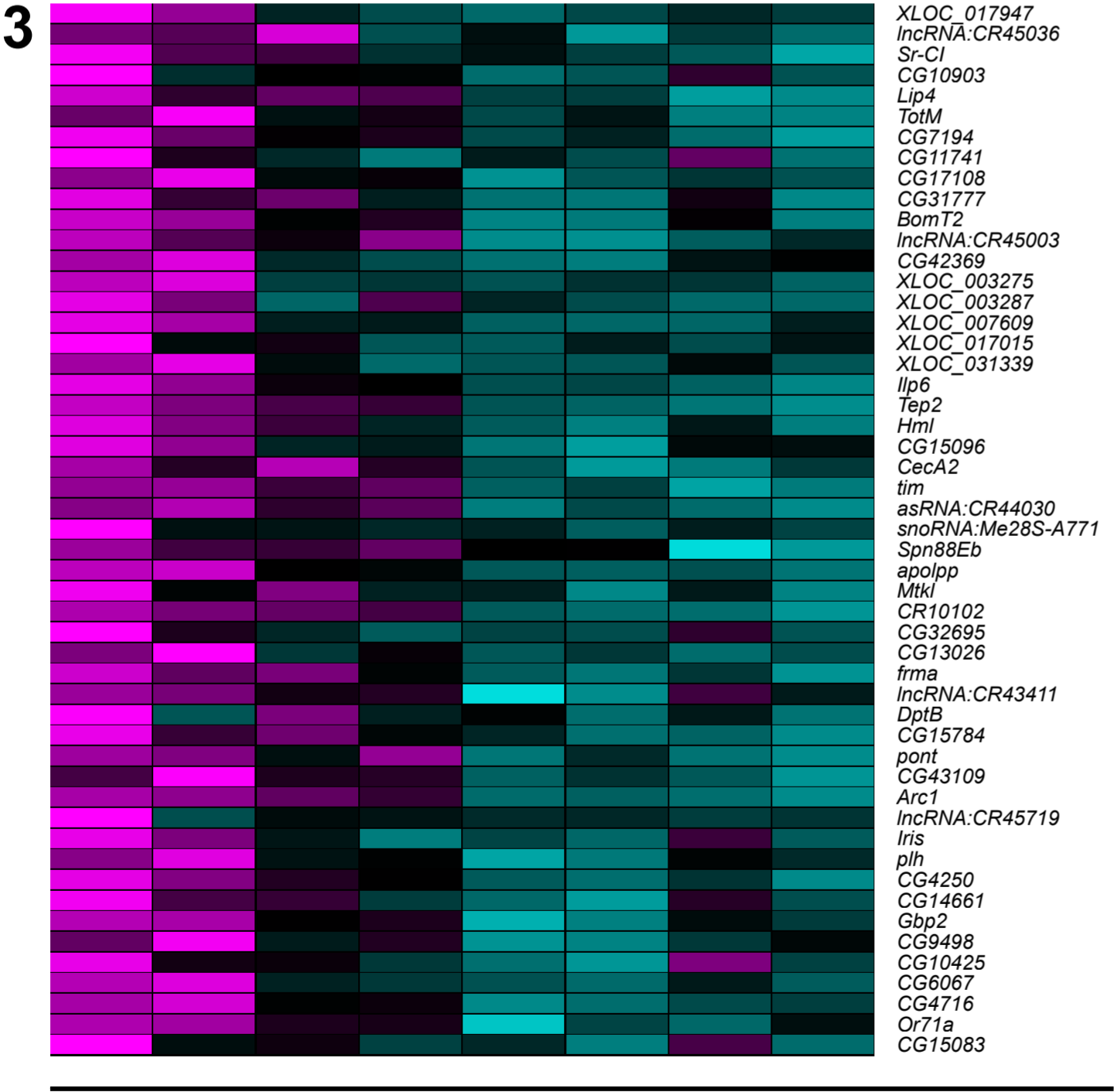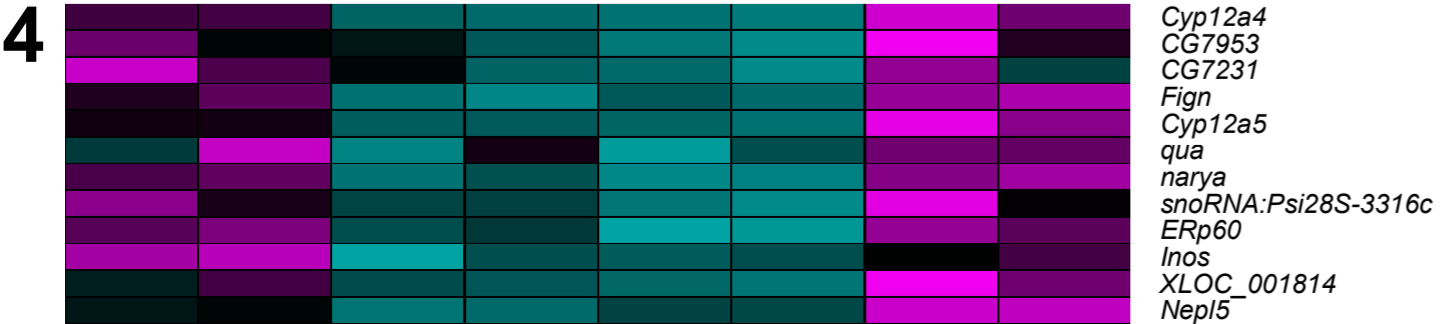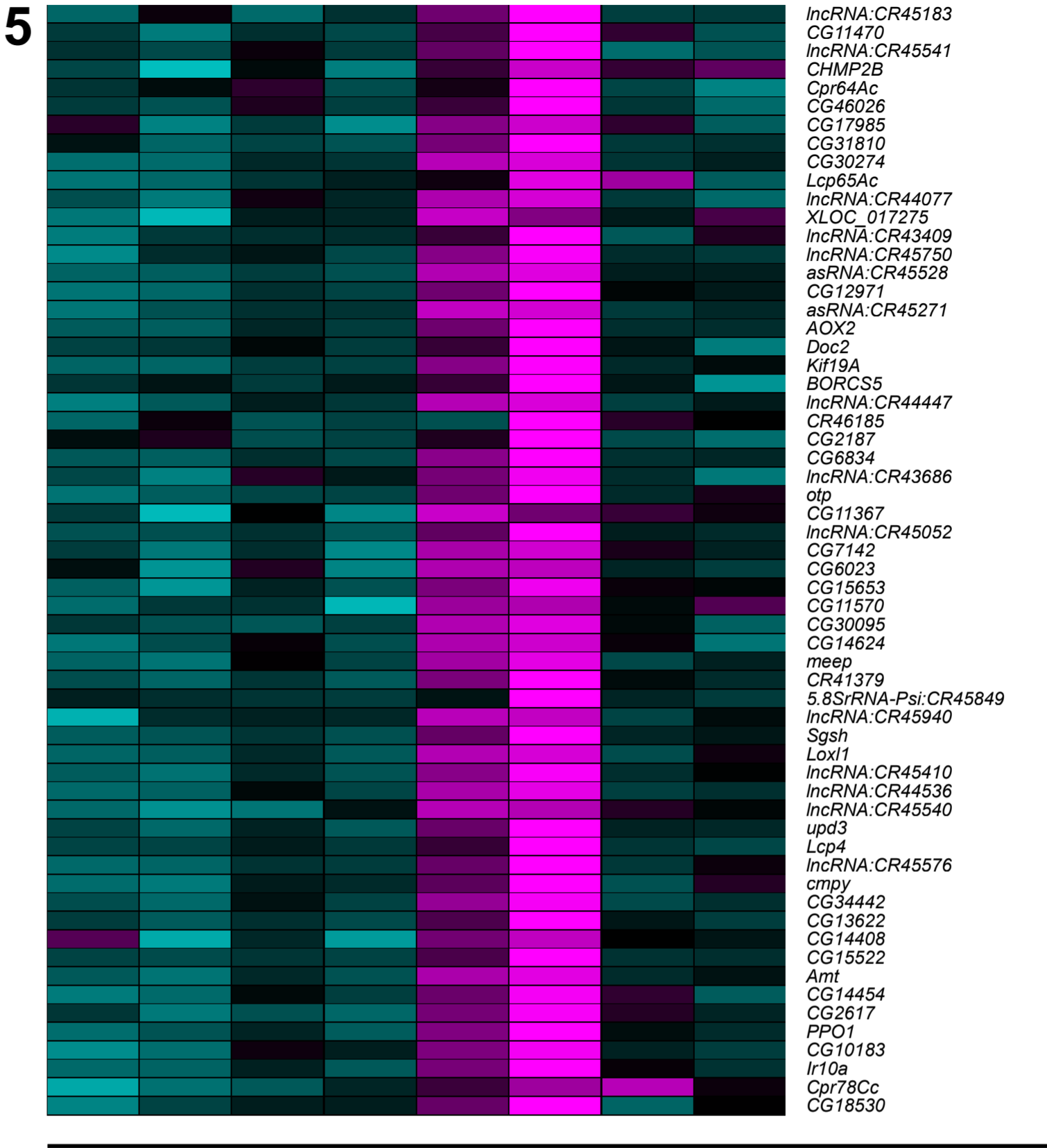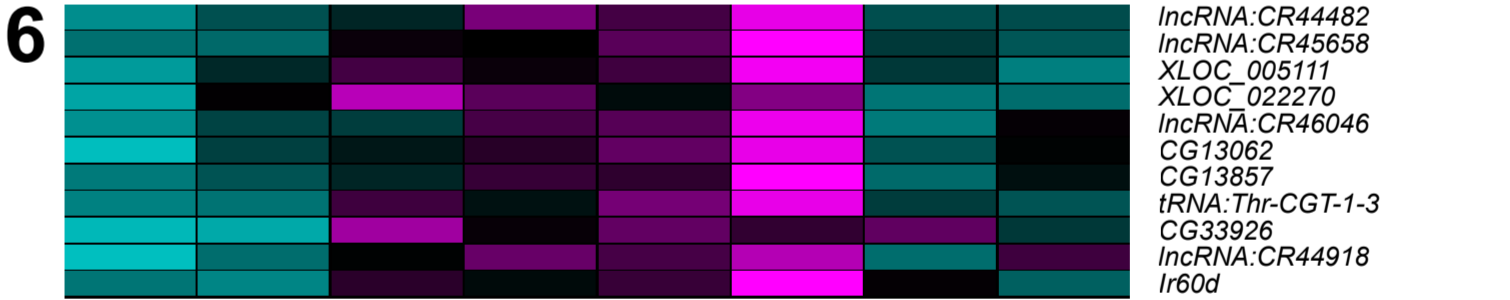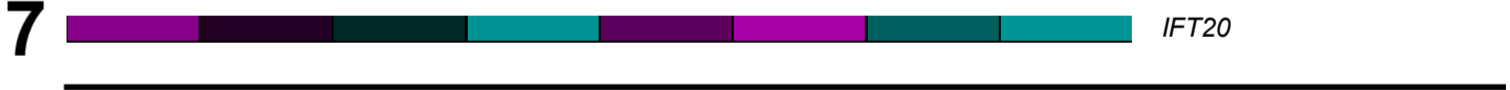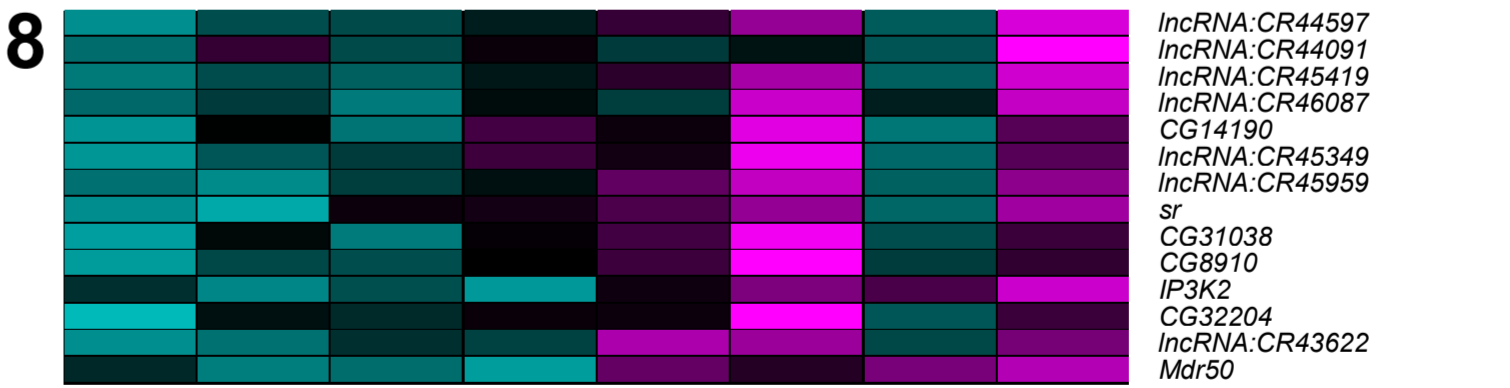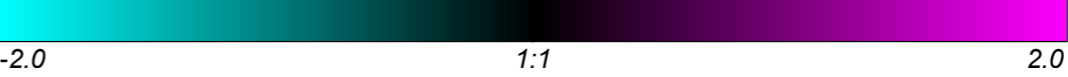

Supplement: Supplementary file 11 — Additional file 11: Figure S4. K-means clusters. K-means clusters derived from genes and NTRs with differential expression in a global transcriptomic analysis using genes significant for the Line or Line×Sex terms (BH-FDR < 0.1). Uhg4 is shown in bold and indicated with a star symbol. Magenta indicates a relatively higher degree of expression, blue indicates a relatively lower degree of expression. [file 12864_2022_8972_MOESM11_ESM.pdf]
