# Supplementary material for: Pleiotropic fitness effects of the lncRNA Uhg4 in Drosophila melanogaster
Source: BMC Genomics. 2022 Nov 30;23:781. doi: 10.1186/s12864-022-08972-0 (PMC9710044; doi:10.1186/s12864-022-08972-0)
Supplement: Supplementary file 10 — Additional file 10: Figure S3. Non-monotonic relationship between raw and Benjamini-Hochberg adjusted p–values. (A) raw p-values plotted against number of tests; (B) BH-FDR thresholds on raw p-values. Raw and adjusted p-values are shown in blue and red, respectively. “i”: rank, “m”: number of tests. See corresponding Table S2. [file 12864_2022_8972_MOESM10_ESM.pdf]

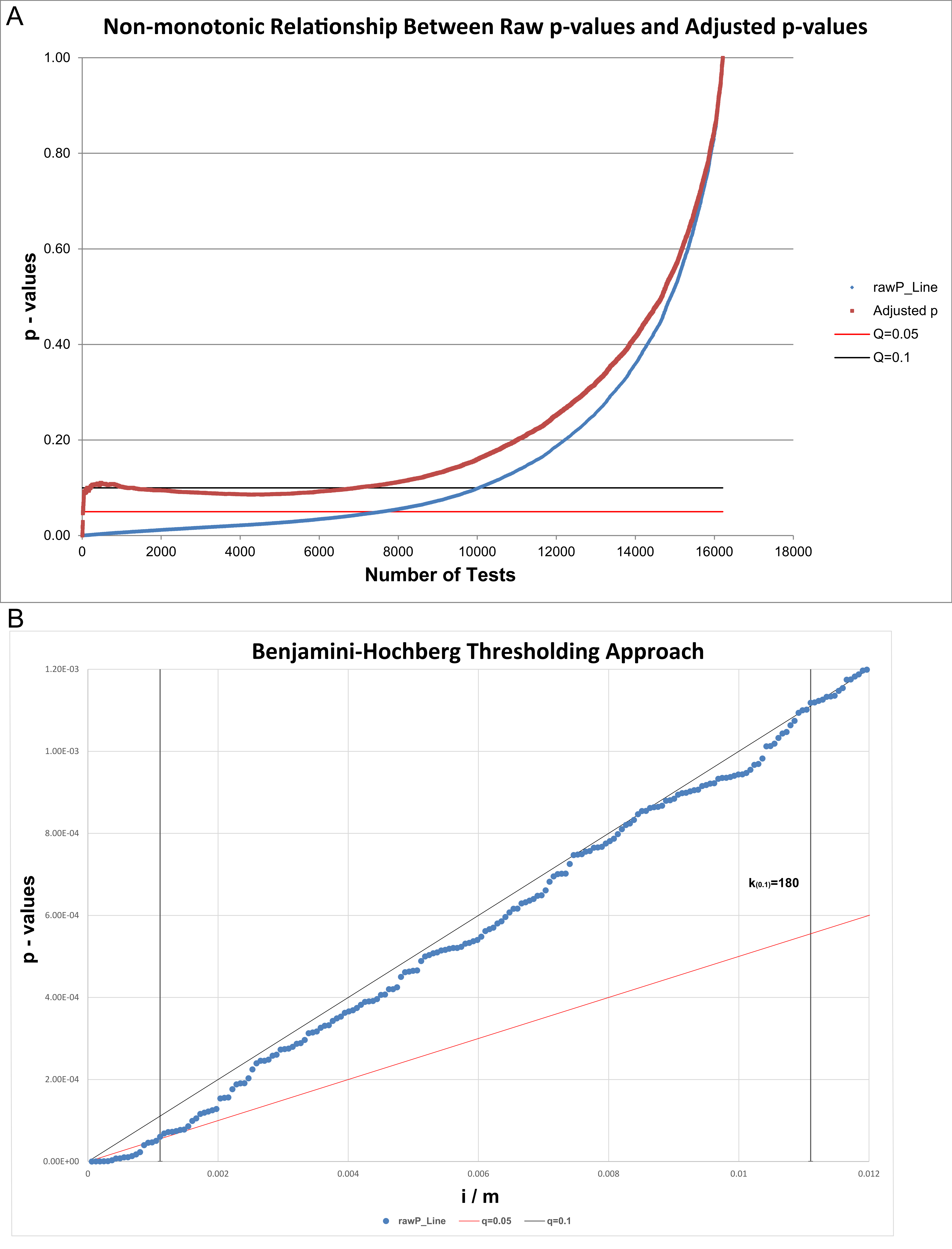
A
Non-monotonic Relationship Between Raw p-values and Adjusted p-values
p - values
1.00
0.80
0.60
0.40
0.20
0.00
0
2000
4000
6000
8000
10000
12000
14000
16000
18000
Number of Tests
rawP_Line
Adjusted p
Q=0.05
Q=0.1
B
Benjamini-Hochberg Thresholding Approach
p - values
1.20E-03
1.00E-03
8.00E-04
6.00E-04
4.00E-04
2.00E-04
0.00E+00
0
0.002
0.004
0.006
0.008
0.01
0.012
i / m
k(0.1)=180
rawP_Line
q=0.05
q=0.1
