# Supplementary material for: Pleiotropic fitness effects of the lncRNA Uhg4 in Drosophila melanogaster
Source: BMC Genomics. 2022 Nov 30;23:781. doi: 10.1186/s12864-022-08972-0 (PMC9710044; doi:10.1186/s12864-022-08972-0)
Supplement: Supplementary file 12 — Additional file 12: Figure S5. Additional interaction networks. Interaction networks containing physical and genetic interactions with genes with a significant Line effect. (A) Networks generated from an input of 20 differentially expressed genes/NTRs (BH-FDR < 0.05) including neighbors within at least 1 degree. (B) Networks generated from an input of 180 coregulated genes (BH-FDR < 0.1) including neighbors within at least 2 degrees. Annotation is based on enriched Gene Ontology terms. Dark green indicates the genes in the input data set and light green indicates interaction neighbors. Names are Drosophila gene symbols. See Table S4. [file 12864_2022_8972_MOESM12_ESM.pdf]

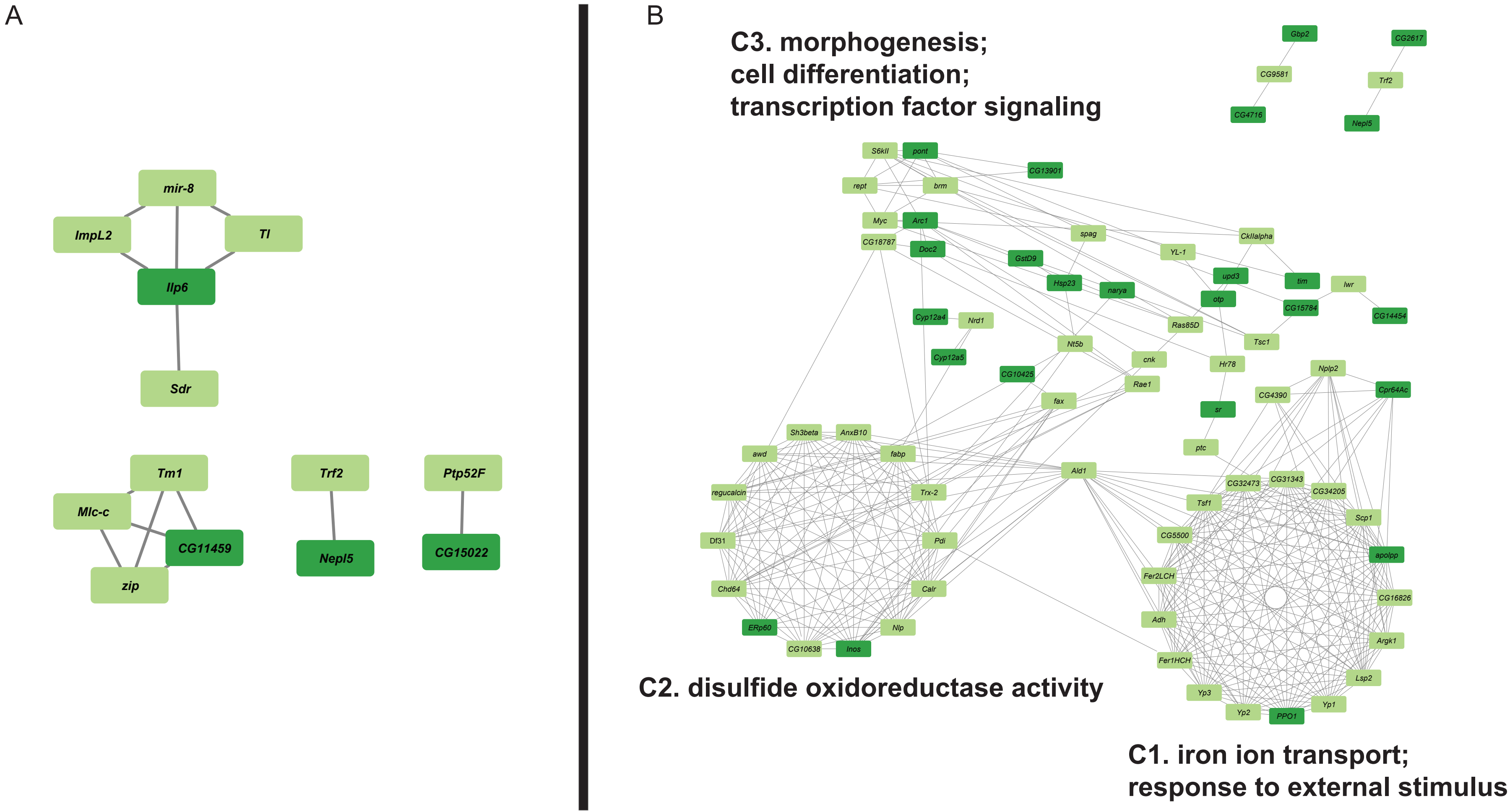
A
mir-8
ImpL2
Tl
Ilp6
Sdr
Tm1
Mlc-c
CG11459
zip
Trf2
Nepl5
Ptp52F
CG15022
B
C3. morphogenesis;
cell differentiation;
transcription factor signaling
Gbp2
CG9581
CG4716
CG2617
Trf2
Nepl5
S6kII
pont
CG13901
rept
brm
Myc
Arc1
spag
CkIIalpha
CG18787
Doc2
GstD9
YL-1
upd3
tim
Iwr
Hsp23
narya
otp
CG15784
CG14454
Cyp12a4
Nrd1
Ras85D
Tsc1
Nt5b
Cyp12a5
cnk
Hr78
CG10425
Rae1
Nplp2
fax
CG4390
Cpr64Ac
sr
Sh3beta
AnxB10
awd
fabp
ptc
Ald1
regucalcin
Trx-2
CG32473
CG31343
CG34205
Tsf1
Scp1
CG5500
Df31
Pdi
apolpp
Fer2LCH
Chd64
Calr
CG16826
Adh
ERp60
Nlp
CG10638
Inos
Argk1
Fer1HCH
Lsp2
Yp3
Yp2
PPO1
Yp1
C2. disulfide oxidoreductase activity
C1. iron ion transport;
response to external stimulus
